# Oral administration of tartrazine (E102) accelerates the incidence and the development of 7,12-dimethylbenz(a) anthracene (DMBA)-induced breast cancer in rats

**DOI:** 10.1186/s12906-021-03490-0

**Published:** 2021-12-31

**Authors:** Stéphane Zingue, Elisabeth Louise Ndjengue Mindang, Florence Charline Awounfack, Abel Yanfou Kalgonbe, Moustapha Mohamet Kada, Dieudonné Njamen, Derek Tantoh Ndinteh

**Affiliations:** 1 Department of Medical and Biomedical Engineering, Higher Technical Teachers’ Training College, University of Yaoundé 1, P.O. Box 886, Ebolowa, Cameroon; 2 Department of Life and Earth Sciences, Higher Teachers’ Training College, University of Maroua, P.O. Box 55, Maroua, Cameroon; 3 Centre for Natural Product Research, Department of Chemical Sciences, University of Johannesburg, P.O. Box 17011, Doornfontein, Johannesburg 2028 South Africa; 4 Department of Animal Biology and Physiology, Faculty of Science, University of Yaoundé 1, P.O. Box 812, Yaounde, Cameroon

**Keywords:** Tartrazine, Breast cancer, 7,12 dimethylbenz(a) anthracene, alpha-fetoprotein, CA 15–3, Oxidant

## Abstract

**Background:**

Despite the considerable advances made in the treatment of cancer, it remains a global threat. Tartrazine (E102) is a synthetic dye widely used in food industries; it has recently been shown to induce oxidative stress (a well known risk factor of cancer) in rat tissues. The present work therefore aimed to assess the impact of a regular consumption of tartrazine on the incidence of breast cancer in rats.

**Methods:**

Forty (40) Wistar rats aged 55 to 60 days were randomly assigned into 5 groups (*n* = 8) including two groups serving as normal controls and receiving distilled water (NOR) or tartrazine (NOR + TARZ). The three remaining groups were exposed to the carcinogen DMBA (50 mg/kg) and treated for 20 weeks with either distilled water (DMBA), tartrazine 50 mg/kg (DMBA + TARZ) or a natural dye (DMBA + COL). The parameters evaluated were the incidence, morphology and some biomarkers (CA 15–3, estradiol and α-fetoprotein) of breast cancer. The oxidative status and histomorphology of the tumors were also assessed.

**Results:**

A regular intake of tartrazine led to an early incidence of tumors (100% in rats that received TARZ only vs 80% in rats that received DMBA only), with significantly larger tumors (*p* < 0.001) (mass = 3500 mg/kg and volume = 4 cm^3^). The invasive breast carcinoma observed on the histological sections of the animals of the DMBA + TARZ group was more developed than those of the DMBA group. The increase in serum α-fetoprotein (*p* < 0.05) and CA 15–3 (*p* < 0.01) levels corroborate the changes observed in tumors. The presence of oxidative activity in animals of the DMBA + TARZ group was confirmed by a significant decrease (*p* < 0.001) in the activity of antioxidant enzymes (SOD and catalase) as well as the level of GSH and increase in the level of MDA compared to the rats of the DMBA and NOR groups.

**Conclusion:**

Tartrazine therefore appears to be a promoter of DMBA-induced breast tumorigenesis in rats through its oxidative potential. This work encourages further studies on the mechanisms of action of tartrazine (E102) and its limits of use.

**Supplementary Information:**

The online version contains supplementary material available at 10.1186/s12906-021-03490-0.

## Background

Cancer is a heterogeneous group of diseases characterized by a multi-stage development of considerable complexity including uncontrolled proliferation, migration, invasion and metastasis [1]. According to Bray et al. [2], 18.1 million new cancer cases were diagnosed with 9.6 million deaths in 2018. Breast cancer is the second most frequently diagnosed cancer in the world with 2.1 million new cases and 626,679 deaths recorded in 2018 [2]. It is therefore a major public health problem for both developing and developed countries [3]. In Cameroon, it ranks 1st among women with more than 3000 new cases diagnosed each year and the majority (80%) of these cases are detected at advanced stages, leading to poor survival at 5 years post-diagnosis [4]. The etiology of breast cancer is still poorly understood, however, several risk factors such as age, hormonal factors (estrogen), environmental factors (Polycyclic aromatic hydrocarbons-PAHs) as well as a family history of cancer have been statistically correlated with this cancer [5]. PAHs including 7,12-dimethylbenz(a)anthracene (DMBA) are environmental cancer initiators from anthropogenic activities which are chemically very stable and can last long in the environment [6]. Once introduced into the body, they are generally metabolized into epoxides that can react with DNA and produce PAH-DNA adducts which are responsible for many human breast tumors [7]. In addition, lifestyle habits and nutrition have also been reported as risk factors for breast cancer [8].

In recent decades, the global food industry makes use of increasing amounts of natural and synthetic food additives. In addition, foods can contain harmful and potentially toxic additives that humans ignore [9]. Food additives include several classes one of which are food dyes, which aim to make food more attractive to consumers [10]. Unfortunately, several of them have been reported to be mutagenic, genotoxic and even carcinogenic [10]. Tartrazine (E102) is one of the most widely used synthetic food dyes in the food industry [11]. It is mainly found in fruit juices, drinks, ice cream, cookies, candies, chocolates, sauces and mustard. It is also used to wrap cold cuts and confectionery products [11]. According to literature, tartrazine could be implicated in allergies, tumor diseases, mutagenic and genotoxicity as well as neuro-behavioral disorders [12, 13]. It is known to induce adverse effects in the pancreas and kidneys and increase the number of kidney tumors in laboratory animals as well as cause chromosomal damage [14–16]. Mpountoukas et al. [17] also showed that tartrazine has the potential to be toxic to human lymphocytes in vitro by binding directly to DNA. Recent studies showed that regular intake of tartrazine increase oxidative stress in various tissues of Wistar rats [18, 19]. Considering the fact that mutagenic and genotoxicity; DNA adducts formation and oxidative effect are all individually known as risk factors of breast cancer, we hypothesized that the regular intake of tartrazine could promote the development of breast tumor once exposed to a carcinogen. This study therefore aimed at evaluating the impact of a regular intake of tartrazine on the development of breast cancer induced by the environmental carcinogen DMBA in Wistar rats. For this, the parameters evaluated were the incidence, morphology and some biomarkers (CA 15–3, estradiol and α-fetoprotein) of breast cancer. The histological analysis of the tumors was also assessed to determine whether or not the coadministration of DMBA plus tartrazine would modify carcinogenic effect of the DMBA on breast.

## Methods

### Chemical substances

The 7,12-dimethylbenz(a)anthracene (DMBA) (purity ≥95%) and tartrazine were obtained from Sigma-Aldrich® (Standford, Germany). The natural dye made from corn starch was obtained at the slaughterhouse market (Maroua, Cameroon). The anesthetics, diazepam (Valium® 10 mg/2 mL) and ketamine (Ketamine hypochloride 50 mg/mL) were obtained from Roche (Fontenay-sous-Bois, France) and Rotex Medica (Tritau, Germany), respectively. ELISA kits for determining alpha-fetoprotein, CA 15–3 and estradiol levels were obtained from Elabscience® (Willich, Germany).

### Experimental animals

Forty (40) prepubertal Wistar rats (*Rattus norvegicus*) aged 41 to 51 days at the start of the experiment and weighing between 70 and 85 g were obtained from the breeding facility of the Animal Physiology Laboratory of the University of Yaoundé 1. These rats were distributed 8 per group in plastic cages at room temperature in the animal house of the Department of Biological Sciences, Faculty of Sciences, University of Maroua where the study was conducted. They had free access to water and were fed with standard rat chow containing: corn meal (36.6%), bone meal (14.5%), cotton seed meal (7.3%), fish (4.8%), cooking salt (0.3%) and vegetable oil.

### Ethical consideration

Housing and animal treatments were approved by the Joint Institutional Review Board Animal & Human Bioethics of the Faculty of Science (University of Yaounde 1), which adopted the directives established by the European Union on the care of animals (EEC Council 86/609).

### Study design

#### Induction of breast cancer

Breast cancer was induced according to the method of Mefegue et al. [20]. Briefly, 50 mg/kg BW of DMBA dissolved in 1 mL of olive oil was thoroughly sonicated and injected subcutaneously (*s.c*) onto the right inguinal mammary gland of the pubescent rats (55–60 days) to induce mammary tumors. Alternatively, normal animals were given olive oil only.

#### Treatment of animals

The dose of tartrazine used in this study was derived from the work of Das and Mukherjee [21], who showed that tartrazine is non-mutagenic and non-genotoxic at doses of 50, 100 and 200 mg/kg BW. The smallest safe dose (50 mg/kg) was therefore chosen to assess its possible promoter effects on the occurrence of breast cancer in female rats exposed to DMBA. For this to be done, 40 female Wistar rats aged 41 to 51 days were acclimatized for 07 days, afterward the rats were randomly assigned into five groups of 8 animals each as follows: Two normal control groups given distilled water (NOR) or tartrazine (NOR + TARZ), respectively. The other 3 groups were exposed to DMBA and received distilled water (DMBA), tartrazine (DMBA + TARZ) and a natural corn starch dye (DMBA + COL). Treatment was performed by gavage a week before exposure to DMBA and thereafter, it continued for 20 weeks. The animals were weighed weekly and palpated twice a week to detect tumor. The moribund rats were sacrificed under anesthesia and for those which died during the experiment autopsy were performed and all parameters have been recorded. At the end of treatment, all the surviving animals were fasted for 12 h, weighed and sacrificed by decapitation under anesthesia consisting of a mixture of ketamine (10 mg/kg BW, *i.p.*) and diazepam (50 mg/kg BW, *i.p.*).

Blood was collected in dry tubes and centrifuged at 3000 rpm for 15 min, then stored at 4 °C for subsequent biochemical analyzes. The skin was then dissected to expose the breast tumors which were all removed, counted and weighed. Estrogen target organs (ovaries, uterus, vagina and mammary glands), major breast cancer metastasizing organs (femur, brain, liver and lungs) and certain organs of interest for toxicity studies (spleen, kidneys and adrenal glands) were removed and weighed. All organs were immediately fixed in 10% formalin for histological analysis.

#### Tumor parameters

The tumor incidence (percentage of affected rats per group), relative tumor weight (tumor weight divided per animal body weight) and tumor burden (the cumulative tumor weight of animals in a group) were determined. The tumor size was measured using an electronic caliper (IGAGING®) and tumor volume was calculated using the formula of Kubatka et al. [22]: width 2 × length/2.

#### Preparation of homogenates of mammary glands and tumors

A part of each mammary gland or tumor was cut, weighed and crushed using Potter teflon-glass on ice using sodium phosphate buffer (0.1 M; pH 7.5) to afford a 20% final homogenate. After centrifugation at 3000 rpm for 15 min at 4 °C, the supernatant was stored at − 20 °C for the determination of total protein level and oxidative status in mammary gland/tumor.

#### Biochemical and hematological analysis

Different hematological parameters were evaluated using a MINDRAY BC-2800 Auto Hematology Analyzer from Shenzhen Mindray Bop-medical Electronics Co., Ltd. These parameters included: white blood cell (WBC) count, lymphocytes, monocytes, granulocytes, red blood cell (RBC) count, hematocrit, hemoglobin, mean corpuscular volume (MCV), mean corpuscular hemoglobin (MCH), mean corpuscular hemoglobin concentration (MCHC) and platelets.

The quantitative determination of estradiol, Alpha-fetoprotein (AFP) and breast cancer biomarker CA 15–3 levels in sera was done using enzyme-linked immunosorbent assay (ELISA) following the manufacturer’s instructions (Monobind Inc.®, California, USA). The estimation of the total protein levels was performed following the methods described by Gonal et al. [23]. Oxidative stress parameters likesuperoxide dismutase (SOD) activity, malondialdehyde (MDA) level, catalase activity and GSH level were measured following the methods of Misra [24], Wilbur et al. [25], Sinha [26] and Ellman [27], respectively.

### Histological analysis

Histopathological changes in mammary glands and tumors were determined by using 5-μm tissue sections of paraffin-embedded organs stained with hematoxylin and eosin. The images were captured using the complete Zeiss equipment consisting of a microscope Axioskop 40 connected to a computer where the images were transferred and analyzed with the MRGrab 1.0 and Axio Vision 3.1 softwares, all provided by Zeiss (Hallbermoos, Germany). The breast tumors were classified using the histopathologic criteria from Russo and Russo [28].

### Statistical analysis

Analysis of variance (ANOVA) followed by Dunnett’s post test for multiple comparisons were used for the various statistical analyzes using GraphPad Prism version 5.00 software. All the animals were included in the analysis and comparison was made between different control and treated groups. The data obtained were expressed as the mean ± standard error of the mean (SEM) and the difference was considered significant at a probability level of 5% (*p* < 0.05).

## Results

### Effects of tartrazine on the incidence of tumors

**Fig. 1:**
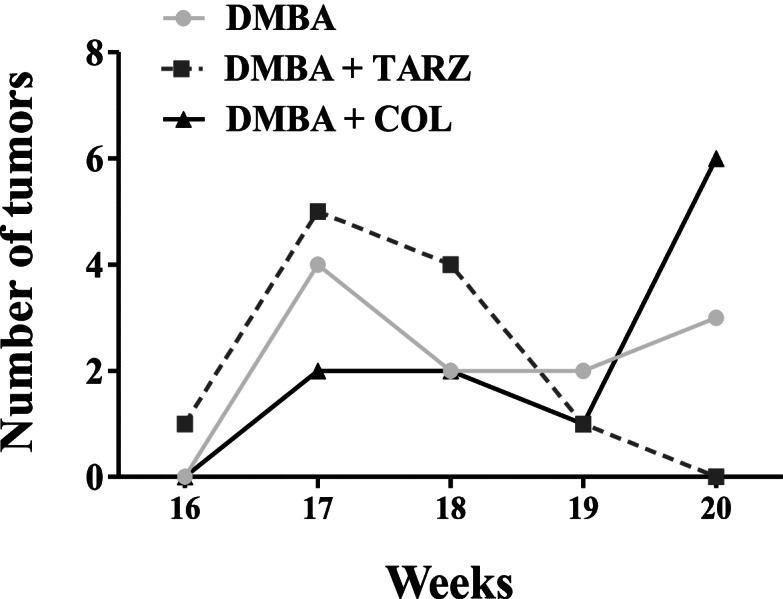
Effects of tartrazine on the incidence of tumors. DMBA = Animals serving as negative control receiving distilled water; DMBA + TARZ = Animals receiving tartrazine at a dose of 50 mg/kg; DMBA + COL = Animals receiving a natural dye at a dose of 50 mg/kg. Dots represent means ± SEM (*n* = 8). All animals were exposed to a single dose of DMBA (50 mg/kg)

During the 20 weeks of this experiment, at least one death was observed in each group. The highest death rate (2 rats out of 8) was recorded in the DMBA + TARZ group. The first tumors were observed in the DMBA + tartrazine group during the 16th week post-exposure to DMBA (Fig. 1). The following week, several tumors were observed in all the groups treated with DMBA: 5 tumors in rats of the DMBA + TARZ group, 4 tumors in animals of the DMBA group and 3 in animals of the DMBA + COL group. At the 19th week, an incidence of tumors of 100% was noted in the DMBA + tartrazine group compared to the DMBA group (with an incidence of 80%). At the end of the 20 weeks of observation, all groups had a 100% incidence of tumors.

### Effects of tartrazine on tumors

**Fig. 2:**
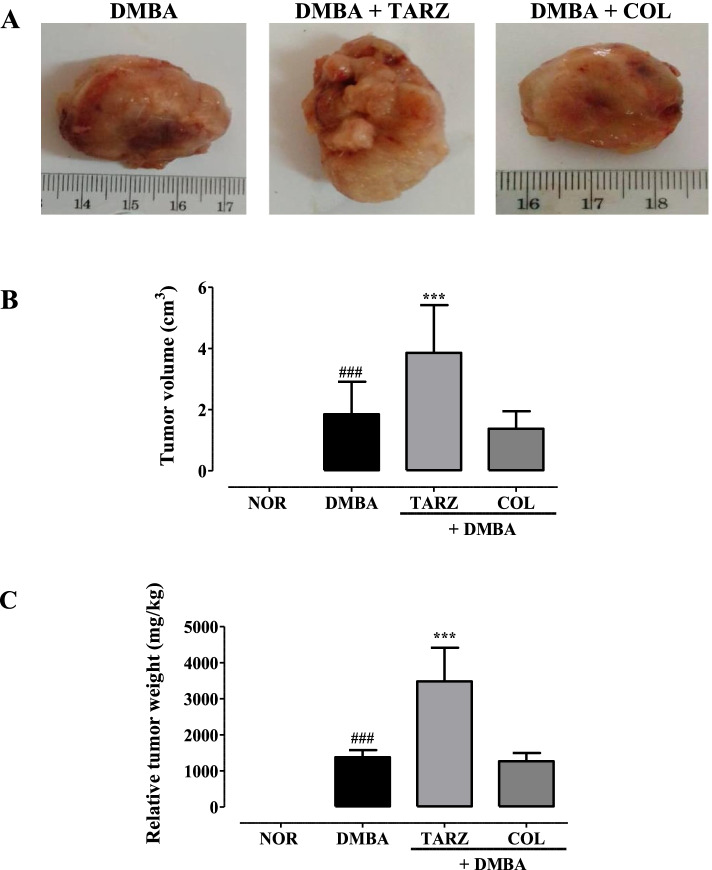
Effects of tartrazine on tumor morphology (A), tumor volume (B) and tumor weight (C). NOR = Animals serving as a normal control receiving distilled water; DMBA = Animals serving as negative control receiving distilled water; DMBA + TARZ = Animals receiving tartrazine at a dose of 50 mg/kg; DMBA + COL = Animals receiving a natural dye at a dose of 50 mg/kg. Dots represent means ± SEM (*n* = 8). All animals were exposed to a single dose of DMBA (50 mg/kg). ### *p* < 0.001 compared to the NOR group; *** *p* < 0.001 compared to the DMBA group

The administration of tartrazine (50 mg/kg) in rats administered with DMBA (50 mg/kg) led to the development significantly (*p* < 0.001) larger tumors compared to those observed in the DMBA group (Fig. 2); with a relative weight ~ 3500 mg/kg *v.s.* ~ 1500 mg/kg and a tumor volume of ~ 4 cm^3^ *v.s.* ~ 2 cm^3^. No significant difference was observed between animals in the DMBA and DMBA + COL groups as well as those of NOR and NOR + TARZ groups.

### Effects on some breast cancer biomarkers

**Fig. 3:**
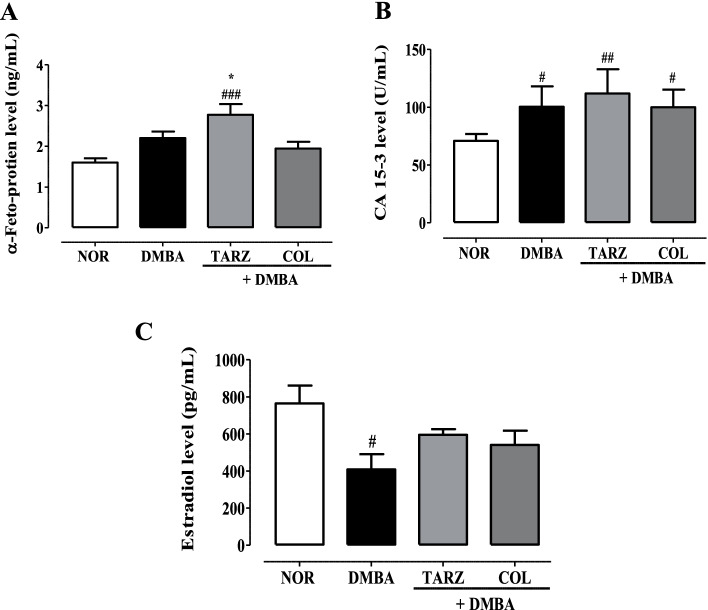
Effects of tartrazine on serum α-fetoprotein (A), CA 15–3 (B) and estradiol (C) levels. NOR = Animals serving as a normal control receiving distilled water; DMBA = Animals serving as negative control receiving distilled water; DMBA + TARZ = Animals receiving tartrazine at a dose of 50 mg/kg; DMBA + COL = Animals receiving a natural dye at a dose of 50 mg/kg. The points represent the means ± ESM (n = 8). All animals were exposed to a single dose of DMBA (50 mg/kg). #*p* < 0.05 compared to the NOR group; * *p* < 0.05, ** *p* < 0.01 *** *p* < 0.001 compared to the DMBA group

The level of α-fetoprotein in the different experimental groups are illustrated in Fig. 3 A. No statistical difference was observed between the Normal (NOR), DMBA and DMBA + COL groups. However, animals exposed to DMBA and treated with tartrazine (DMBA + TRAZ) had a significant (*p* < 0.001) high level of α-fetoprotein as compared to the DMBA group. Fig. 3 B shows the levels of CA 15–3 in different experimental groups. Higher levels of CA 15–3 were observed in all groups of animals treated with DMBA compared to animals in the normal group. However, the level of the CA15–3 level was most pronounced in DMBA + TARZ as compared to the DMBA group.

Figure 3 C revealed that administration of DMBA alone induced a significant decrease (*p* < 0.05) in serum estradiol level compared to the normal group. Furthermore, no significant difference was observed in the different treated groups.

### Effects on some parameters of oxidative stress

Table 1 depicts the effect of treatment with tartrazine and natural dye on the oxidative status of mammary glands and tumors. There was a decrease in GSH level as well as the activity of the antioxidant enzymes (SOD and catalase), although only significant with catalase activity (*p* < 0.05) in DMBA group as compared to NOR. Animals that received tartrazine and DMBA showed a significant (*p* < 0.001) decrease in the activity of antioxidant enzymes (SOD and catalase) as well as the GSH level compared to rats belonging to the DMBA and normal (NOR) groups. Moreover, after 20 weeks of treating rats exposed to DMBA with tartrazine, there was a significant (*p* < 0.001) increase in lipid peroxidation, evidenced by an increase in the level of MDA as compared to animals of the DMBA and normal groups. There was no significant difference in the activity of the antioxidant enzymes SOD and catalase as well as in the levels of GSH and MDA between the groups treated with DMBA alone, DMBA + COL and normal animals.

**Table 1:** Effects of tartrazine and the natural dye (COL) on some parameters of oxidative stress in the mammary glands and tumors

	NOR	NOR + TARZ	DMBA	DMBA + TARZ	DMBA + COL
• MDA (mM/mg de protéines)	4.37 ± 0.13	4.7 ± 0.92	4.4 ± 0.24	6.2 ± 0.53**##	4.92 ± 0.51
• SOD (unité/mg de protéines)	108.5 ± 0.74	116.1 ± 0.31	95.1 ± 0.13	74.9 ± 0.22**##	111.52 ± 0.54
• Catalase (mM de H_2_O_2_/min/mg de protéines)	0.71 ± 0.02	0.68 ± 0.04	0.46 ± 0.02#	0.23 ± 0.02**##	0.48 ± 0.03
• GSH (mM/mg de protéines)	0.01 ± 0.0017	0.01 ± 0.0004	0.008 ± 0.0002	0.006 ± 0.000**##**	0.016 ± 0.0004

*NOR* Animals serving as normal control receiving distilled water, *NOR + TARZ* Animals receiving tartrazine only, *DMBA* Animals serving as negative control receiving distilled water, *DMBA + TARZ* Animals receiving tartrazine at a dose of 50 mg/kg BW, *DMBA + COL* Animals receiving a natural dye at a dose of 50 mg/kg. #*p* < 0.05; ## *p* < 0.01 compared to the NOR group; ** p < 0.01 compared to the DMBA group

### Effects on the histopathology of the mammary gland and tumor

**Fig. 4:**
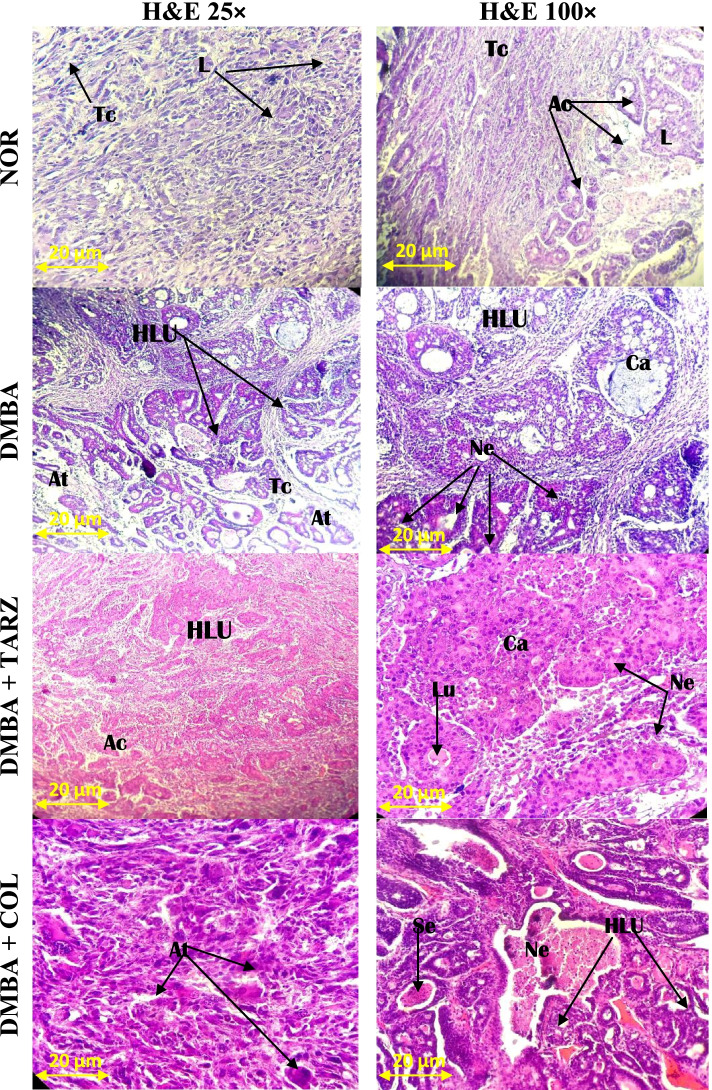
Photomicrographs (H&E × 25 and H&E × 100) of cross section of mammary glands and tumors after exposure to different substances. NOR = Animals serving as a normal control receiving distilled water; DMBA = Animals serving as negative control receiving distilled water; DMBA + TARZ = Animals receiving tartrazine at a dose of 50 mg/kg; DMBA + COL = Animals receiving a natural dye at a dose of 50 mg/kg. Ca = carcinoma; At = adipose tissue; Se = Eosinophilic secretion, L = lobule; HLU = Hypertrophied lobular unit

All animals exposed to DMBA at a dose of 50 mg/kg developed tumors after 20 weeks. In fact the histological sections showed mononuclear mammary ductal cells surrounded by abundant adipose tissues (Fig. 4). In contrast, the tumor sections of all groups exposed to DMBA showed mammary carcinoma displaying polymorphic epithelial cells with evidence of excessive proliferation leading to the obstruction of intralobular acini.

The histological sections of animals co-treated with TARZ + DMBA presented a pronounced alteration in the microarchitecture of the mammary gland with a high Scarff-Bloom-Richardson grade cribriform ductal carcinoma, with excessive proliferation and signs of inflammation as compared to the DMBA and DMBA + COL groups (Fig. 4).

### Effects on body mass and the mass of some fresh organs

All animals which were exposed to DMBA showed a significant decrease (*p* < 0.05) in body weight at the end of 20 weeks of experimentation compared to the normal group (Table 2). In addition, a significant increase in lungs (*p* < 0.05), kidneys (*p* < 0.05) and spleen (*p* < 0.001) wet weight was observed in animals exposed only to DMBA compared to the normal animals. Animals exposed to DMBA and treated with tartrazine (*p* < 0.001) or natural dye (*p* < 0.01) significantly prevented the increase in spleen weight induced by DMBA. Moreover, regular intake of natural corn-based dye protected (*p* < 0.05) against the increase in relative lung mass induced by DMBA.

**Table 2:** Effects of tartrazine and the natural dye (COL) on body mass and the relative weight of some organs

	NOR	NOR+ TARZ	DMBA	DMBA + TARZ	DMBA + COL
**Body weight (g)**					
Initial	135.5 ± 6.7	129.8 ± 5.4	121.7 ± 6.9	124.4 ± 4.6	126.4 ± 11.9
Final	203.5 ± 6.1	195.4 ± 2.9	179.6 ± 4.2#	182.4 ± 9.6#	182.8 ± 8.2#
**Organ weight (%)**					
Uterus	0.23 ± 0.03	0.27 ± 0.05	0.36 ± 0.05	0.25 ± 0.01	0.25 ± 0.04
Liver	2.98 ± 0.1	2.96 ± 0.15	3.19 ± 0.17	3.04 ± 0.13	2.84 ± 0.11
Lung	0.6 ± 0.07	0.58 ± 0.06	0.79 ± 0.07#	0.67 ± 0.06	0.60 ± 0.03*
Spleen	0.24 ± 0.01	0.22 ± 0.01	0.42 ± 0.04###	0.28 ± 0.006***	0.29 ± 0.03**
Adrenals	0.03 ± 0.003	0.04 ± 0.004	0.03 ± 0.009	0.02 ± 0.006	0.03 ± 0.003
Kidney	0.54 ± 0.03	0.53 ± 0.01	0.60 ± 0.02#	0.61 ± 0.01	0.59 ± 0.02
Femur	0.4 ± 0.01	0.25 ± 0.01	0.27 ± 0.06	0.26 ± 0.01	0.37 ± 0.03
Brain	0.62 ± 0.01	0.73 ± 0.02	0.77 ± 0.17	0.87 ± 0.063	0.54 ± 0.2
Ovaries	0.07 ± 0.004	0.08 ± 0.01	0.08 ± 0.01	0.067 ± 0.01	0.06 ± 0.01

*NOR* Animals serving as normal control receiving distilled water, *NOR + TARZ* Animals receiving tartrazine only, *DMBA* Animals serving as negative control receiving distilled water, *DMBA + TARZ* Animals receiving tartrazine at a dose of 50 mg/kg, *DMBA + COL* Animals receiving a natural dye at a dose of 50 mg/kg. #*p* < 0.05; ### *p* < 0.001 compared to the NOR group; *p < 0.05; **p < 0.01; ***p < 0.001 compared to the DMBA group

No difference in body weights and relative organ weights was observed between the normal animals which took tartrazine and those which received the vehicle (distilled water) at the end of the 20 weeks study period (Table 2).

## Discussion

Breast cancer is a real public health problem around the world despite the significant advances made in treatment and care [3]. It is a heterogeneous and multifactorial disease in which lifestyle habits and nutrition play an essential role. Food additives are known to be potentially toxic to certain functions of the body [29]. Tartrazine (E102) is a synthetic dye widely used in the food and pharmaceutical industries [30]. It is an endocrine disruptor that has been reported to cause oxidative stress in rats [18, 19]. The present study therefore aimed to assess the impact of tartrazine on the incidence of DMBA-induced breast cancer in rats. The DMBA-induced mammary tumor model used in this study is one of models of breast cancer widely used in rodents. Indeed this model is acclaimed for the histological and molecular similarities with human mammary cancer. DMBA is an environmental chemical carcinogen that induces genotoxicity via its hepatic 3,4-dihydrodiol-1,2-epoxide metabolites and the free radicals they generate [31]. The results obtained from this work showed that the incidence of mammary tumors was higher in animals exposed to DMBA and treated with tartrazine (100%) compared to animals exposed only to DMBA (80%), suggesting that tartrazine increases the incidence of mammary tumors in animals in which cancer has been initiated. These results are in line with studies which have reported the genotoxic effects of tartrazine through its capability to bind to DNA or by its biotransformation into sulfanilic acid and aminopyrazolone, which can generate free radicals which in turn induce oxidative stress [32]. In line with these effects, an increase in relative tumor weight and tumor volume was noted in DMBA + TARZ rats compared to animals exposed to DMBA only, suggesting an accelerating effect of tartrazine on mammary tumorogenesis. Indeed, Datta and Lundin-Schiller [33] reported significant proliferative effects of tartrazine on estrogen-dependent breast cancer cells T47D, which is in accordance with our observations. Moreover, Axon et al. [34] demonstrated that tartrazine can transactivate estrogen receptor alpha (ERα) in the estrogen-dependent breast cancer line MCF-7 with an effective concentration of 160 nM. Estrogens are potent promoters of estrogen-dependent cancers such as breast cancer.

The α-fetoprotein is a major 69 Kda glycoprotein of fetal serum, produced first by the yolk sac early in gestation and then by the liver. It is used in the screening for fetal malformations and in the detection of maternal tumors [35]. It is involved in the regulation of proliferation, differentiation and survival of different cell types, both embryonic and tumor [36]. As far as it is concerned, CA 15–3 is a biomarker of breast cancer overexpressed during cell proliferation [31]. Estradiol is the main female sex hormone of a steroid nature, involved in breast carcinogenesis as initiator and promoter [37]. In this study, an increase in serum α-fetoprotein, CA 15–3 and estradiol levels was observed in animals exposed to DMBA and tartrazine as compared to the DMBA rats. These results are consistent with the higher tumor incidence, tumor weight and tumor volume observed in DMBA + TARZ group as compared to the DMBA group. These high levels of CA 15–3 and α-fetoprotein portray the establishment of cancer and are in agreement with the observations of Nguedia et al. [31]. Several studies have demonstrated the undeniable role of oxidative stress in the carcinogenesis of the human breast; this generally results from an established imbalance between pro-oxidant and anti-oxidant [38]. Free radicals and reactive oxygen species have received particular attention especially in experimental medicine and in Biology: This is because of their role in the etiology of various diseases including cancer. It has been shown that the harmful effects of reactive oxygen species on cells can be abrogated by plants containing antioxidant compounds. Indeed, SOD is a metalloprotein which represents one of the first enzymatic lines of defense against oxidative stress by ensuring the elimination of the superoxide anion by a disproportionation reaction [39]. MDA, for its part, is one of the derivatives of lipid peroxidation and a biomarker of oxidative stress [40]. In this work, animals that received both tartrazine and DMBA showed a significant decrease in antioxidant enzyme activity (SOD and catalase) as well as GSH level and increased in MDA level compared to both DMBA and normal rats. The increased level of MDA and the decrease in SOD and catalase activity suggest that tartrazine induces oxidative stress. These results corroborate the observations of several authors [18, 19] who observed similar results in Wistar rats. It is well known that DMBA induces part of its deleterious effects on DNA via the formation of mutagenic free radicals; therefore the oxidative activity of tartrazine would potentiate the effect of DMBA which would in turn accelerate carcinogenesis in rats exposed to both DMBA and tartrazine. In line with this hypothesis, the histological analysis of the microarchitecture of the mammary glands of animals treated with tartrazine and DMBA showed more pronounced alterations with excessive proliferation, compared to the DMBA group. These results are in line with those obtained on the tumor incidence and tumor weight as well as tumor volume, and corroborate the observation of Saxema and schama [41]; who have shown that the administration of tartrazine induces histopathological changes.

No significant difference was observed between animals in the DMBA and DMBA + COL groups in the all the assessed parameters, suggesting the inability of this natural corn starch dye to protect against breast cancer. Although some work has reported the anticancer potential of corn leaves (*Zea Mays*) [42], no study has yet demonstrated the anticancer effects of its seeds. The decrease in the body weight of the animals observed in this study would be associated with the toxicity of DMBA which induces a state of morbidity in the animals, which can lead to anorexia. These results are in line with those previously obtained in our research unit [20, 30]. Organ mass is a good indicator of the harmful effects of drugs and/or any other toxicant [43]. The increase in the relative weight of the spleen in DMBA rats may be due hemorrhagic lesions and immuninotoxicity induced by DMBA [20]. In addition, Khayyal et al. [13] after 49 days of oral administration with tartrazine at 7.5 and 15 mg/kg, reported genotoxicity at the level of detoxification organs such as the kidney and the liver.

## Conclusion

It emerges from this study that the regular intake of tartrazine leads to an early incidence of tumors (100% in DMBA + TARZ group vs 80% in DMBA group). In addition, significantly (*p* < 0.001) larger tumor weights (3500 mg/kg and volume = 4 cm^3^) were observed in DMBA + TARZ animals compared to animals in the DMBA group. The invasive mammary carcinoma observed in animals in the DMBA + TARZ group displayed excessive proliferation than those in the DMBA group. The increase in serum α-fetoprotein (*p* < 0.05) and CA 15–3 (*p* < 0.01) levels corroborate the results observed on tumor morphology. Tartrazine which, is widely consumed by humans and produced in various forms by the food industry, therefore appears to be a promoter of mammary tumorigenesis induced by DMBA in rats via its oxidative potential. This work encourages further studies on the mechanisms of action of tartrazine (such as programmed cell death, the evidence for cell division, transactivation of estrogen receptors) and its limits of use.

## Supplementary Information

**Additional file 1.** The ARRIVE Guidelines Checklist

## Data Availability

The datasets used and/or analysed during the current study available from the corresponding author on reasonable request.

## Abbreviations

AFP: Alpha-fetoprotein;
ANOVA: Analysis of variance
BW: body weight
CA 15–3: cancer antigen 15–3
COL: Natural dye
DMBA: 7,12 dimethylbenz(a)anthracene
DNA: Deoxyribonucleic acid
EDTA: ethylenediaminetetraacetic acid
ER: estrogen receptor
Hb: hemoglobin
Ht: hematocrit
Kda: kilodaltons
NOR: Normal
MDA: Malonedialdehide
PAHs: Polycyclic aromatic hydrocarbons
RBC: red blood cell
rpm: run per minute
SEM: standard error on mean
SOD: superoxide dismutase
TARZ: tartrazine
