# Supplementary material for: Oral administration of tartrazine (E102) accelerates the incidence and the development of 7,12-dimethylbenz(a) anthracene (DMBA)-induced breast cancer in rats
Source: BMC Complement Med Ther. 2021 Dec 31;21:303. doi: 10.1186/s12906-021-03490-0 (PMC8720219; doi:10.1186/s12906-021-03490-0)
Supplement: Supplementary file 1 — Additional file 1. The ARRIVE Guidelines Checklist [file 12906_2021_3490_MOESM1_ESM.doc]

**The ARRIVE Guidelines Checklist**

|  | **ITEM** | **RECOMMENDATION** | | **Section/ Paragraph** | |
| --- | --- | --- | --- | --- | --- |
| **Title** | **1** | Provide as accurate and concise a description of the content of the article as possible | | Oral administration of tartrazine (E102) accelerates the incidence and the development of 7,12-dimethylbenz(a)anthracene (DMBA)-induced breast cancer in rats | |
| **Abstract** | **2** | Provide an accurate summary of the background, research objectives, including details of the species or strain of animal used, key methods, principal findings and conclusions of the study. | | **Background**: Despite the considerable advances made in the treatment of cancer, it remains a global threat. Tartrazine (E102) is a synthetic dye widely used in food industries; it has recently been shown to induce oxidative stress (a well known risk factor of cancer) in rat tissues. The present work therefore aimed to assess the impact of a regular consumption of tartrazine on the incidence of breast cancer in rats.  **Methods**: Forty (40) Wistar rats aged 55 to 60 days were randomly assigned into 5 groups (n = 8) including two groups serving as normal controls and receiving distilled water (NOR) or tartrazine (NOR + TARZ). The three remaining groups were exposed to the carcinogen DMBA (50 mg/kg) and treated for 20 weeks with either distilled water (DMBA), tartrazine 50 mg/kg (DMBA + TARZ) or a natural dye (DMBA + COL). The parameters evaluated were the incidence, morphology and some biomarkers (CA 15-3, estradiol and α-fetoprotein) of breast cancer. The oxidative status and histomorphology of the tumors were also assessed.  **Results**: A regular intake of tartrazine led to an early incidence of tumors (100% in rats that received TARZ only *vs* 80% in rats that received DMBA only), with significantly larger tumors (*p* < 0.001) (mass = 3500 mg/kg and volume = 4 cm^3^). The invasive breast carcinoma observed on the histological sections of the animals of the DMBA + TARZ group was more developed than those of the DMBA group. The increase in serum α-fetoprotein (*p* < 0.05) and CA 15-3 (*p* < 0.01) levels corroborate the changes observed in tumors. The presence of oxidative activity in animals of the DMBA + TARZ group was confirmed by a significant decrease (*p* < 0.001) in the activity of antioxidant enzymes (SOD and catalase) as well as the level of GSH and increase in the level of MDA compared to the rats of the DMBA and NOR groups.  **Conclusion**: Tartrazine therefore appears to be a promoter of DMBA-induced breast tumorigenesis in rats through its oxidative potential. This work encourages further studies on the mechanisms of action of tartrazine (E102) and its limits of use. | |
| **INTRODUCTION** | | | | | |
| **Background** | **3** | **a.** Include sufficient scientific background (including relevant references to previous work) to understand the motivation and context for the study, and explain the experimental approach and rationale. | | Cancer is a heterogeneous group of diseases characterized by a multi-stage development of considerable complexity including uncontrolled proliferation, migration, invasion and metastasis [1]. According to Bray et al. [2], 18.1 million new cancer cases were diagnosed with 9.6 million deaths in 2018. Breast cancer is the second most frequently diagnosed cancer in the world with 2.1 million new cases and 626,679 deaths recorded in 2018 [2]. It is therefore a major public health problem for both developing and developed countries [3]. In Cameroon, it ranks 1^st^ among women with more than 3,000 new cases diagnosed each year and the majority (80%) of these cases are detected at advanced stages, leading to poor survival at 5 years post-diagnosis [4]. The etiology of breast cancer is still poorly understood, however, several risk factors such as age, hormonal factors (estrogen), environmental factors (Polycyclic aromatic hydrocarbons-PAHs) as well as a family history of cancer have been statistically correlated with this cancer [5]. PAHs including 7,12-dimethylbenz(a)anthracene (DMBA) are environmental cancer initiators from anthropogenic activities which are chemically very stable and can last long in the environment [6]. Once introduced into the body, they are generally metabolized into epoxides that can react with DNA and produce PAH-DNA adducts which are responsible for many human breast tumors [7]. In addition, lifestyle habits and nutrition have also been reported as risk factors for breast cancer [8]. | |
|  |  | **b.** Explain how and why the animal species and model being used can address the scientific objectives and, where appropriate, the study’s relevance to human biology. | | According to literature, tartrazine could be implicated in allergies, tumor diseases, mutagenic and genotoxicity as well as neuro-behavioral disorders [12, 13]. It is known to induce adverse effects in the pancreas and kidneys and increase the number of kidney tumors in laboratory animals as well as cause chromosomal damage [14, 15, 16]. | |
| **Objectives** | **4** | Clearly describe the primary and any secondary objectives of the study, or specific hypotheses being tested. | | This study therefore aimed at evaluating the impact of a regular intake of tartrazine on the development of breast cancer induced by the environmental carcinogen DMBA in Wistar rats. For this, the parameters evaluated were the incidence, morphology and some biomarkers (CA 15-3, estradiol and α-fetoprotein) of breast cancer. The histological analysis of the tumors was also assessed to determine whether or not the coadministration of DMBA plus tartrazine would modify carcinogenic effect of the DMBA on breast. | |
| **METHODS** | | | | | |
| **Ethical statement** | **5** | Indicate the nature of the ethical review permissions, relevant licences (e.g. Animal [Scientific Procedures] Act 1986), and national or institutional guidelines for the care and use of animals, that cover the research. | | Housing and animal treatments were approved by the Joint Institutional Review Board Animal & Human Bioethics of the Faculty of Science (University of Yaounde 1), which adopted the directives established by the European Union on the care of animals (EEC Council 86/609). | |
| **Study design** | **6** | For each experiment, give brief details of the study design including:  **a.**The number of experimental and control groups.  **b.** Any steps taken to minimise the effects of subjective bias when allocating animals to treatment (e.g. randomisation procedure) and when assessing results (e.g. if done, describe who was blinded and when).  **c**. The experimental unit (e.g. a single animal, group or cage of animals). A time-line diagram or flow chart can be useful to illustrate how complex study designs were carried out. | | Breast cancer was induced according to the method of Mefegue *et al*. [20]. Briefly, 50 mg/kg BW of DMBA dissolved in 1 mL of olive oil was thoroughly sonicated and injected subcutaneously (*s.c*) onto the right inguinal mammary gland of the pubescent rats (55-60 days) to induce mammary tumors. Alternatively, normal animals were given olive oil only.  The dose of tartrazine used in this study was derived from the work of Das and Mukherjee [21], who showed that tartrazine is non-mutagenic and non-genotoxic at doses of 50, 100 and 200 mg/kg BW. The smallest safe dose (50 mg/kg) was therefore chosen to assess its possible promoter effects on the occurrence of breast cancer in female rats exposed to DMBA. For this to be done, 40 female Wistar rats aged 41 to 51 days were acclimatized for 07 days, afterward the rats were randomly assigned into five groups of 8 animals each as follows: Two normal control groups given distilled water (NOR) or tartrazine (NOR + TARZ), respectively. The other 3 groups were exposed to DMBA and received distilled water (DMBA), tartrazine (DMBA + TARZ) and a natural corn starch dye (DMBA + COL). | |
| **Experimental procedures** | **7** | For each experiment and each experimental group, including controls, provide precise details of all procedures carried out. For example:  **a.** How (e.g. drug formulation and dose, site and route of administration, anaesthesia and analgesia used [including monitoring], surgical procedure, method of euthanasia). Provide details of any specialist equipment used, including supplier(s).  **b.** When (e.g. time of day).  **c.** Where (e.g. home cage, laboratory, water maze).  **d.** Why (e.g. rationale for choice of specific anaesthetic, route of administration, drug dose used). | | Treatment was performed by gavage a week before exposure to DMBA and thereafter, it continued for 20 weeks. The animals were weighed weekly and palpated twice a week to detect tumor. The moribund rats were sacrificed under anesthesia and for those which died during the experiment autopsy were performed and all parameters have been recorded. At the end of treatment, all the surviving animals were fasted for 12 hours, weighed and sacrificed by decapitation under anesthesia consisting of a mixture of ketamine (10 mg/kg BW, *i.p.*) and diazepam (50 mg/kg BW, *i.p.*).  Blood was collected in dry tubes and centrifuged at 3000 rpm for 15 min, then stored at 4 °C for subsequent biochemical analyzes. The skin was then dissected to expose the breast tumors which were all removed, counted and weighed. Estrogen target organs (ovaries, uterus, vagina and mammary glands), major breast cancer metastasizing organs (femur, brain, liver and lungs) and certain organs of interest for toxicity studies (spleen, kidneys and adrenal glands) were removed and weighed. All organs were immediately fixed in 10% formalin for histological analysis. | |
| **Experimental animals** | **8** | **a**. Provide details of the animals used, including species, strain, sex, developmental stage (e.g. mean or median age plus age range) and weight (e.g. mean or median weight plus weight range).  **b.** Provide further relevant information such as the source of animals, international strain nomenclature, genetic modification status (e.g. knock-out or transgenic), genotype, health/immune status, drug or test naïve, previous procedures, etc. | | Forty (40) prepubertal Wistar rats (*Rattus norvegicus*) aged 41 to 51 days at the start of the experiment and weighing between 70 and 85 g were obtained from the breeding facility of the Animal Physiology Laboratory of the University of Yaoundé 1. | |
| **Housing and husbandry** | **9** | Provide details of:  a. Housing (type of facility e.g. specific pathogen free [SPF]; type of cage or housing; bedding material; number of cage companions; tank shape and material etc. for fish).  b. Husbandry conditions (e.g. breeding programme, light/dark cycle, temperature, quality of water etc for fish, type of food, access to food and water, environmental enrichment).  c. Welfare-related assessments and interventions that were carried out prior to, during, or after the experiment. | | These rats were distributed 8 per group in plastic cages at room temperature in the animal house of the Department of Biological Sciences, Faculty of Sciences, University of Maroua where the study was conducted.  They had free access to water and were fed with standard rat chow containing: corn meal (36.6%), bone meal (14.5%), cotton seed meal (7.3%), fish (4.8%), cooking salt (0.3%) and vegetable oil. | |
| **Sample size** | **10** | **a.** Specify the total number of animals used in each experiment, and the number of animals in each experimental group. b. Explain how the number of animals was arrived at. Provide details of any sample size calculation used.  c. Indicate the number of independent replications of each experiment, if relevant. | | Forty (40) prepubertal Wistar rats aged 41 to 51 days at the start of the experiment and weighing between 70 and 85 g were obtained from the breeding facility of the Animal Physiology Laboratory of the University of Yaoundé 1. | |
| **Allocating animals to experimental groups** | **11** | **a.** Give full details of how animals were allocated to experimental groups, including randomisation or matching if done.  b. Describe the order in which the animals in the different experimental groups were treated and assessed. | | In order to assess the impact of tartrazine (E102) on the occurrence of breast cancer in female rats exposed to DMBA, 40 female Wistar rats aged 41 to 51 days were acclimatized for 07 days, afterward the rats were randomly assigned into five groups of 8 animals each as follows: Two normal control groups given distilled water (NOR) or tartrazine (NOR + TARZ), respectively. The other 3 groups were exposed to DMBA and received distilled water (DMBA), tartrazine (DMBA + TARZ) and a natural corn starch dye (DMBA + COL). Treatment was performed by gavage a week before exposure to DMBA and thereafter, it continued for 20 weeks. The animals were weighed weekly and palpated twice a week to detect tumor. | |
| **Experimental outcomes** | **12** | Clearly define the primary and secondary experimental outcomes assessed (e.g. cell death, molecular markers, behavioural changes). | | For this, the parameters evaluated were the incidence, morphology and some biomarkers (CA 15-3, estradiol and α-fetoprotein) of breast cancer. The histological analysis of the tumors was also assessed to determine whether or not the coadministration of DMBA plus tartrazine would modify carcinogenic effect of the DMBA on breast. | |
| **Statistical methods** | **13** | **a.** Provide details of the statistical methods used for each analysis.  b. Specify the unit of analysis for each dataset (e.g. single animal, group of animals, single neuron).  c. Describe any methods used to assess whether the data met the assumptions of the statistical approach. | | Analysis of variance (ANOVA) followed by Dunnett's post test for multiple comparisons were used for the various statistical analyzes using GraphPad Prism version 5.00 software. All the animals were included in the analysis and comparison was made between different control and treated groups. The data obtained were expressed as the mean ± standard error of the mean (SEM) and the difference was considered signiﬁcant at a probability level of 5% (*p* < 0.05). | |
| **RESULTS** |  | |  | |  |
| **Baseline data** | **14** | | For each experimental group, report relevant characteristics and health status of animals (e.g. weight, microbiological status, and drug or test naïve) prior to treatment or testing. (This information can often be tabulated). | | Forty (40) prepubertal Wistar rats aged 41 to 51 days at the start of the experiment and weighing between 70 and 85 g were obtained from the breeding facility of the Animal Physiology Laboratory of the University of Yaoundé 1. |
| **Numbers analysed** | **15** | | a. Report the number of animals in each group included in each analysis. Report absolute numbers (e.g. 10/20, not 50%^2^).  b. If any animals or data were not included in the analysis, explain why. | | The moribund rats were sacrificed under anesthesia and for those which died during the experiment autopsy were performed and all parameters recorded. All the animals were included in the analysis and comparison was made between different control and treated groups. |
| **Outcomes and estimation** | **16** | | Report the results for each analysis carried out, with a measure of precision (e.g. standard error or confidence interval). | | The data are presented as mean ± standard error of the mean (SEM). |
| **Adverse events** | **17** | | **a.** Give details of all important adverse events in each experimental group.  b. Describe any modifications to the experimental protocols made to reduce adverse events. | | **Not applicable** |
| **DISCUSSION** | | | | | |
| **Interpretation/ scientific implications** | **18** | | **a**. Interpret the results, taking into account the study objectives and hypotheses, current theory and other relevant studies in the literature. | | Breast cancer is a real public health problem around the world despite the significant advances made in treatment and care [3]. It is a heterogeneous and multifactorial disease in which lifestyle habits and nutrition play an essential role. Food additives are known to be potentially toxic to certain functions of the body [29]. Tartrazine (E102) is a synthetic dye widely used in the food and pharmaceutical industries [30]. It is an endocrine disruptor that has been reported to cause oxidative stress in rats [18, 19]. The present study therefore aimed to assess the impact of tartrazine on the incidence of DMBA-induced breast cancer in rats. The DMBA-induced mammary tumor model used in this study is one of models of breast cancer widely used in rodents. Indeed this model is acclaimed for the histological and molecular similarities with human mammary cancer. DMBA is an environmental chemical carcinogen that induces genotoxicity via its hepatic 3,4-dihydrodiol-1,2-epoxide metabolites and the free radicals they generate [31]. The results obtained from this work showed that the incidence of mammary tumors was higher in animals exposed to DMBA and treated with tartrazine (100%) compared to animals exposed only to DMBA (80%), suggesting that tartrazine increases the incidence of mammary tumors in animals in which cancer has been initiated. These results are in line with studies which have reported the genotoxic effects of tartrazine through its capability to bind to DNA or by its biotransformation into sulfanilic acid and aminopyrazolone, which can generate free radicals which in turn induce oxidative stress [32]. In line with these effects, an increase in relative tumor weight and tumor volume was noted in DMBA + TARZ rats compared to animals exposed to DMBA only, suggesting an accelerating effect of tartrazine on mammary tumorogenesis. Indeed, Datta and Lundin-Schiller [33] reported significant proliferative effects of tartrazine on estrogen-dependent breast cancer cells T47D, which is in accordance with our observations. Moreover, Axon *et al.* [34] demonstrated that tartrazine can transactivate estrogen receptor alpha (ERα) in the estrogen-dependent breast cancer line MCF-7 with an effective concentration of 160 nM. Estrogens are potent promoters of estrogen-dependent cancers such as breast cancer.  The α-fetoprotein is a major 69 Kda glycoprotein of fetal serum, produced first by the yolk sac early in gestation and then by the liver. It is used in the screening for fetal malformations and in the detection of maternal tumors [35]. It is involved in the regulation of proliferation, differentiation and survival of different cell types, both embryonic and tumor [36]. As far as it is concerned, CA 15-3 is a biomarker of breast cancer overexpressed during cell proliferation [31]. Estradiol is the main female sex hormone of a steroid nature, involved in breast carcinogenesis as initiator and promoter [37]. In this study, an increase in serum α-fetoprotein, CA 15-3 and estradiol levels was observed in animals exposed to DMBA and tartrazine as compared to the DMBA rats. These results are consistent with the higher tumor incidence, tumor weight and tumor volume observed in DMBA + TARZ group as compared to the DMBA group. These high levels of CA 15-3 and α-fetoprotein portray the establishment of cancer and are in agreement with the observations of Nguedia *et al*. [31]. Several studies have demonstrated the undeniable role of oxidative stress in the carcinogenesis of the human breast; this generally results from an established imbalance between pro-oxidant and anti-oxidant [38]. Free radicals and reactive oxygen species have received particular attention especially in experimental medicine and in Biology: This is because of their role in the etiology of various diseases including cancer. It has been shown that the harmful effects of reactive oxygen species on cells can be abrogated by plants containing antioxidant compounds. Indeed, SOD is a metalloprotein which represents one of the first enzymatic lines of defense against oxidative stress by ensuring the elimination of the superoxide anion by a disproportionation reaction [39]. MDA, for its part, is one of the derivatives of lipid peroxidation and a biomarker of oxidative stress [40]. In this work, animals that received both tartrazine and DMBA showed a significant decrease in antioxidant enzyme activity (SOD and catalase) as well as GSH level and increased in MDA level compared to both DMBA and normal rats. The increased level of MDA and the decrease in SOD and catalase activity suggest that tartrazine induces oxidative stress. These results corroborate the observations of several authors [18, 19] who observed similar results in Wistar rats. It is well known that DMBA induces part of its deleterious effects on DNA via the formation of mutagenic free radicals; therefore the oxidative activity of tartrazine would potentiate the effect of DMBA which would in turn accelerate carcinogenesis in rats exposed to both DMBA and tartrazine. In line with this hypothesis, the histological analysis of the microarchitecture of the mammary glands of animals treated with tartrazine and DMBA showed more pronounced alterations with excessive proliferation, compared to the DMBA group. These results are in line with those obtained on the tumor incidence and tumor weight as well as tumor volume, and corroborate the observation of Saxema and schama [41]; who have shown that the administration of tartrazine induces histopathological changes. |
|  |  |  | b. Comment on the study limitations including any potential sources of bias, any limitations of the animal model, and the imprecision associated with the results^2^. | | No significant difference was observed between animals in the DMBA and DMBA + COL groups in the all the assessed parameters, suggesting the inability of this natural corn starch dye to protect against breast cancer. Although some work has reported the anticancer potential of corn leaves (*Zea Mays*) [42], no study has yet demonstrated the anticancer effects of its seeds. The decrease in the body weight of the animals observed in this study would be associated with the toxicity of DMBA which induces a state of morbidity in the animals, which can lead to anorexia. These results are in line with those previously obtained in our research unit [20, 30]. Organ mass is a good indicator of the harmful effects of drugs and/or any other toxicant [43]. The increase in the relative weight of the spleen in DMBA rats may be due hemorrhagic lesions and immuninotoxicity induced by DMBA [20]. In addition, Khayyal *et al*. [13] after 49 days of oral administration with tartrazine at 7.5 and 15 mg/kg, reported genotoxicity at the level of detoxification organs such as the kidney and the liver. |
|  |  |  | c. Describe any implications of your experimental methods or findings for the replacement, refinement or reduction (the 3Rs) of the use of animals in research. | | **Not applicable** |
| **Generalisability/ translation** | **19** | | Comment on whether, and how, the findings of this study are likely to translate to other species or systems, including any relevance to human biology. | | These results corroborate the observations of several authors [15, 16] who observed similar results in Wistar rats. It is well known that DMBA induces part of its deleterious effects on DNA via the formation of mutagenic free radicals; therefore the oxidative activity of tartrazine would potentiate the effect of DMBA which would in turn accelerate carcinogenesis in rats exposed to both DMBA and tartrazine.  Tartrazine which is widely consumed by humans and produced in various forms by the food industry, therefore appears to be a promoter of mammary tumorigenesis induced by DMBA in rats via its oxidative potential. |
| **Funding** | **20** | | List all funding sources (including grant number) and the role of the funder(s) in the study. | | **Not applicable** |
